# Non-empirical identification of trigger sites in heterogeneous processes using persistent homology

**DOI:** 10.1038/s41598-018-21867-z

**Published:** 2018-02-23

**Authors:** Masao Kimura, Ippei Obayashi, Yasuo Takeichi, Reiko Murao, Yasuaki Hiraoka

**Affiliations:** 1 Photon Factory, Institute of Materials Structure Science, High Energy Accelerator Research Organization (KEK), Tsukuba, Ibaraki, 305–0801 Japan; 2 Department of Materials Structure Science, School of High Energy Accelerator Science, SOKENDAI (The Graduate University for Advanced Studies), Tsukuba, Ibaraki, 305–0801 Japan; 3 Advanced Institute for Materials Research (AIMR), Tohoku University, Sendai, Miyagi 980–8577 Japan; 4 Advanced Technology Research Laboratories, Nippon Steel & Sumitomo Metal Co., Futtsu, Chiba, 293–8511 Japan; 5 Center for Materials research by Information Integration (CMI2), Research and Services Division of Materials Data and Integrated System (MaDIS), National Institute for Materials Science (NIMS), Tsukuba, Ibaraki, 305–0047 Japan; 6 Center for Advanced Intelligence Project, RIKEN, Tokyo, 103–0027 Japan

## Abstract

Macroscopic phenomena, such as fracture, corrosion, and degradation of materials, are associated with various reactions which progress heterogeneously. Thus, material properties are generally determined not by their averaged characteristics but by specific features in heterogeneity (or ‘trigger sites’) of phases, chemical states, etc., where the key reactions that dictate macroscopic properties initiate and propagate. Therefore, the identification of trigger sites is crucial for controlling macroscopic properties. However, this is a challenging task. Previous studies have attempted to identify trigger sites based on the knowledge of materials science derived from experimental data (‘empirical approach’). However, this approach becomes impractical when little is known about the reaction or when large multi-dimensional datasets, such as those with multiscale heterogeneities in time and/or space, are considered. Here, we introduce a new persistent homology approach for identifying trigger sites and apply it to the heterogeneous reduction of iron ore sinters. Four types of trigger sites, ‘hourglass’-shaped calcium ferrites and ‘island’- shaped iron oxides, were determined to initiate crack formation using only mapping data depicting the heterogeneities of phases and cracks without prior mechanistic information. The identification of these trigger sites can provide a design rule for reducing mechanical degradation during reduction.

## Introduction

Trigger sites are specific regions or features of heterogeneity in a material where key reactions initiate and take place in systems. In addition, the macroscopic properties of materials are determined by heterogeneous reactions, such as fractures, corrosion, and degradation. Previous studies have attempted to determine the locations of the trigger sites of heterogeneous processes on the basis of materials-science knowledge derived from experimental data. These ‘empirical’ approaches successfully identified trigger sites in a simple system such as metals. For example, the fracture behaviour of metallic materials has been studied by identifying the links between their microstructure, dislocation mechanisms, and fracture properties^1,2^. Grain boundaries impede dislocation movement, and large-grain microstructures weaken the grain boundaries (trigger sites), deteriorating the mechanical properties of the material^1,2^. These studies generally rely on the structural information provided by experimental techniques such as transmission electron microscopy (TEM) or scanning electron microscopy (SEM). Fundamental materials-science notions such as dislocation theory and micromechanics are used to analyse these microscopic data and empirically identify the trigger sites. However, these empirical approaches become impractical in more complicated systems such as composite materials (e.g. iron ore sinters and carbon fibre reinforced plastics (CFRP)), batteries, and catalysts, where the heterogeneity of the microstructure and/or chemical states are substantially different depending on their locations in a material, and the features evolve during a period of operation. Thus, a conventional analysis of the heterogeneity, such as fractions of co-existing phases averaged over material, is not sufficient, but their distribution or spatial correlation is indispensable.

**Figure 1:**
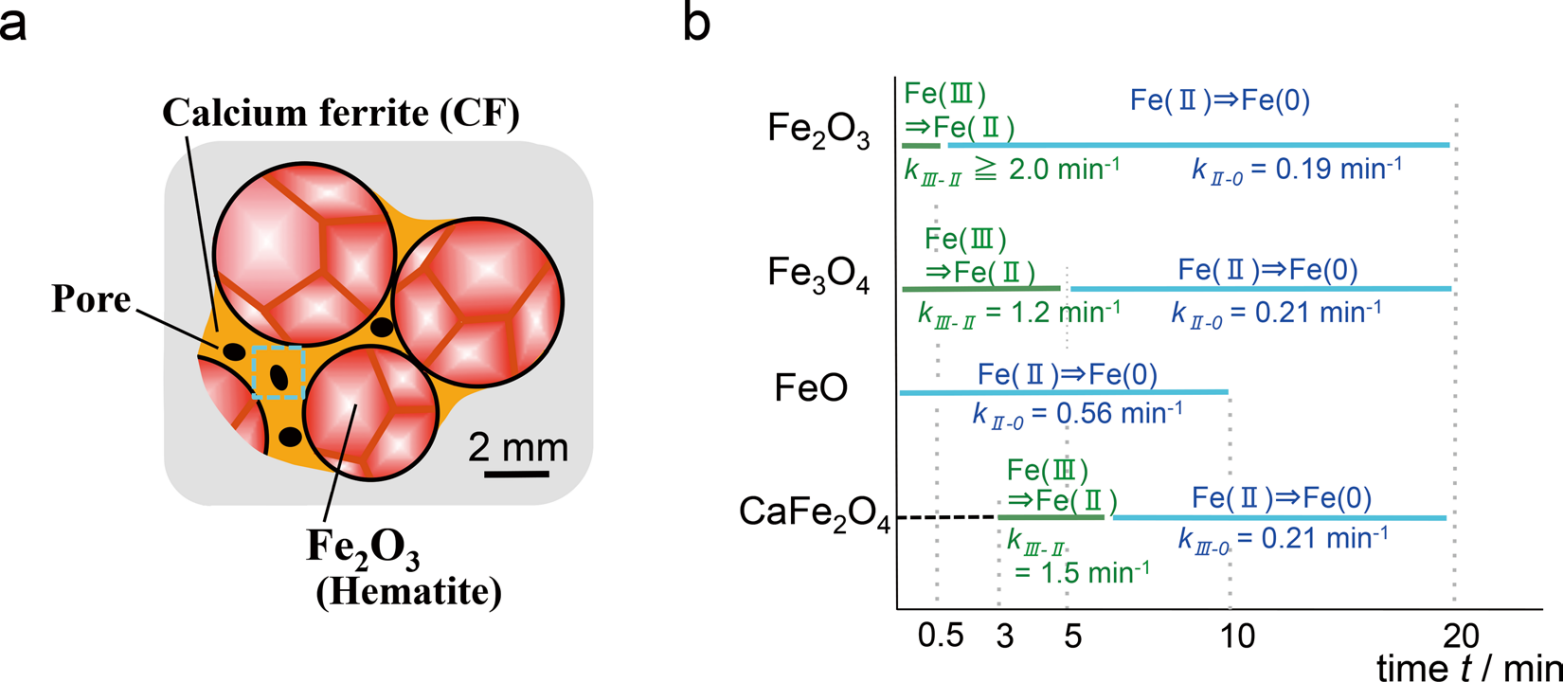
Reduction of iron ore sinters. (**a**) Schematic of an iron ore sinter before reduction. (**b**) Iron reduction rates for the Ca–Fe–O phases corresponding to the main phases in the sinters, which were measured using bulk powder samples in the previous studies^28,29^.

The understanding of heterogeneous processes can be particularly important for materials with industrial applications, such as iron ore sinters. An iron ore sinter is a starting material for iron-making processes and used in blast furnaces in most countries. Iron ores with a weight of 3 × 10^12^ kg are used for the production of sinters every year worldwide, and even a small increase in the reduction efficiency of iron ore sinters has an enormous impact on environmental and energy issues as well as costs. The required property for iron ore sinters is not only a high reducibility but also a low mechanical degradation during reduction in order to support iron ore sinters above themselves (weighing few 10^6^ kg) in a blast furnace with few tens of meter in height. A sinter is formed by liquid sintering at *T* > 1500 K, in which iron oxide grains (mainly α-Fe_2_O_3_) are sintered with bonding layers composed of various types of calcium ferrites^3–5^. Porous networks are then formed during the solidification of the molten Ca–Fe–O (Fig. 1a). The intrinsic differences in the reduction rates of the individual phases (Fig. 1b), together with the effect of the porous structure on the reductive gas flow, control the heterogeneous reduction and—because the reduction involves a large volume decrease—lead to the formation of microcracks^6^. These complex effects complicate the empirical prediction of how the heterogeneous reduction evolves using only data related to the reduction rates of each phase. The determination of the trigger sites of crack formation during reduction is even more difficult because the heterogeneous progress of reduction (and the ensuing increase in the local stress that results in crack formation) is due to both the nature and microstructure of the phases involved. Furthermore, the identification of trigger sites using conventional computational techniques such as finite-element methods to calculate the stress field^7,8^ is not a feasible option. This is because such calculations require parameters such as the Young’s moduli and Poisson’s ratios of all phases as well as the details of the microstructure, and both change from their initial values according to the progress of the process. Because of these difficulties, the trigger sites of crack initiation during reduction have not been identified so far, although they are indispensable information for achieving industrial targets to attain a high reducibility and low mechanical degradation during reduction.

In order to consider differences in heterogeneity in complex systems, we must effectively manage large multi-dimensional datasets in which various types of heterogeneities are observed over multiscales in time and/or space. For example, recent advances in X-ray microscopy (XRM) techniques^9,10^ have enabled the three-dimensional quantification of the heterogeneity of the chemical states and microstructures in a material with spatial resolutions as small as 30 nm. In these cases, the final dataset can reach up gigabytes when we measure X-CT images including a spectrum for each voxel using different X-ray energies. Empirical determination of trigger sites is almost impossible in these cases and the task is even further complicated when only limited information regarding the actual reaction mechanism is available.

Image analysis and machine learning could become a very powerful and effective approach for analysing the large datasets recorded during a heterogeneous reaction. For this approach to be successfully applied to heterogeneous reactions, we must define accurate descriptors of the evolution of the heterogeneity which are closely correlated with the macroscopic properties. However, present techniques^11–15^ do not yet provide good descriptors of the heterogeneity of our interests. A key contribution towards this goal may come from topological data analysis^16,17^.

The rapid development of this research field has produced several tools for analysing the multi-scale data generated in the physical and biological fields^18–25^. A particularly important approach in topological data analysis is persistent homology^26,27^, which takes a multi-scale approach to measure topological features. There are two characteristics—one geometric, assigning a function to a space, and the other algebraic, turning the function into measurements. Thus, persistent homology provides an appropriate descriptor for quantitatively characterizing the evolution of heterogeneity during reactions of our interests in which both the microstructure and chemical states of iron in iron-ore sinters change during reduction and the change of heterogeneity in both is closely correlated with macroscopic properties. Once we accurately describe the evolution of chemical states and microstructures during reduction, machine learning can be used to identify the trigger sites of crack formation caused by their evolution.

Here, we introduce a persistent homology approach for determining the trigger sites of crack initiation in iron ore sinters only using mapping data depicting the heterogeneities of the chemical states and phases involved without prior knowledge such as the reaction mechanism; in so doing, we enable the ‘non-empirical’ identification of trigger sites. The iron ore system is of great importance for industrial applications, as mentioned above, and possesses one of the clearest heterogeneous processes to examine our approach because it is accompanied by a sharp change in the oxidation state of iron in the oxide phase from Fe(III) to Fe(II). The distribution of chemical states (Fe(III) and Fe(II)) evolves heterogeneously according to the progress of reduction^28,29^, and its evolution is expected to be closely related to crack initiation. The trigger sites obtained by the proposed approach without information about the reaction mechanism are discussed in terms of materials-scientific knowledge based on reported experimental data such as the iron reduction rates for the individual iron oxide and Ca–Fe–O phases measured using bulk powder specimens (Fig. 1b)^28,29^.

## Results

### Chemical states and phase mapping

Sinter specimens were prepared by liquid sintering from mixture of iron ore and limestone. Then, the specimens were heated up to 1473 K in a CO/CO_2_ reducing gas atmosphere. Different degrees of reduction were obtained by quenching specimens at different temperatures from 873 K to 1373 K. (see Methods, Section 1 and Supplementary Information, Note S1). The iron oxide states (Fe(III) and Fe(II)) and oxide phases in samples corresponding to different stages of the reduction process were mapped in two dimensions using X-ray absorption near-edge structure (XANES) and X-CT measurements (see Methods, Section 2 and Supplementary Information, Note S2). The bonding region around the iron oxide grains (indicated by the dotted square in Fig. 1a) was mapped with a higher resolution because cracks were considered to form more often in the bonding region than in the oxide grains themselves^3,5^.

**Figure 2:**
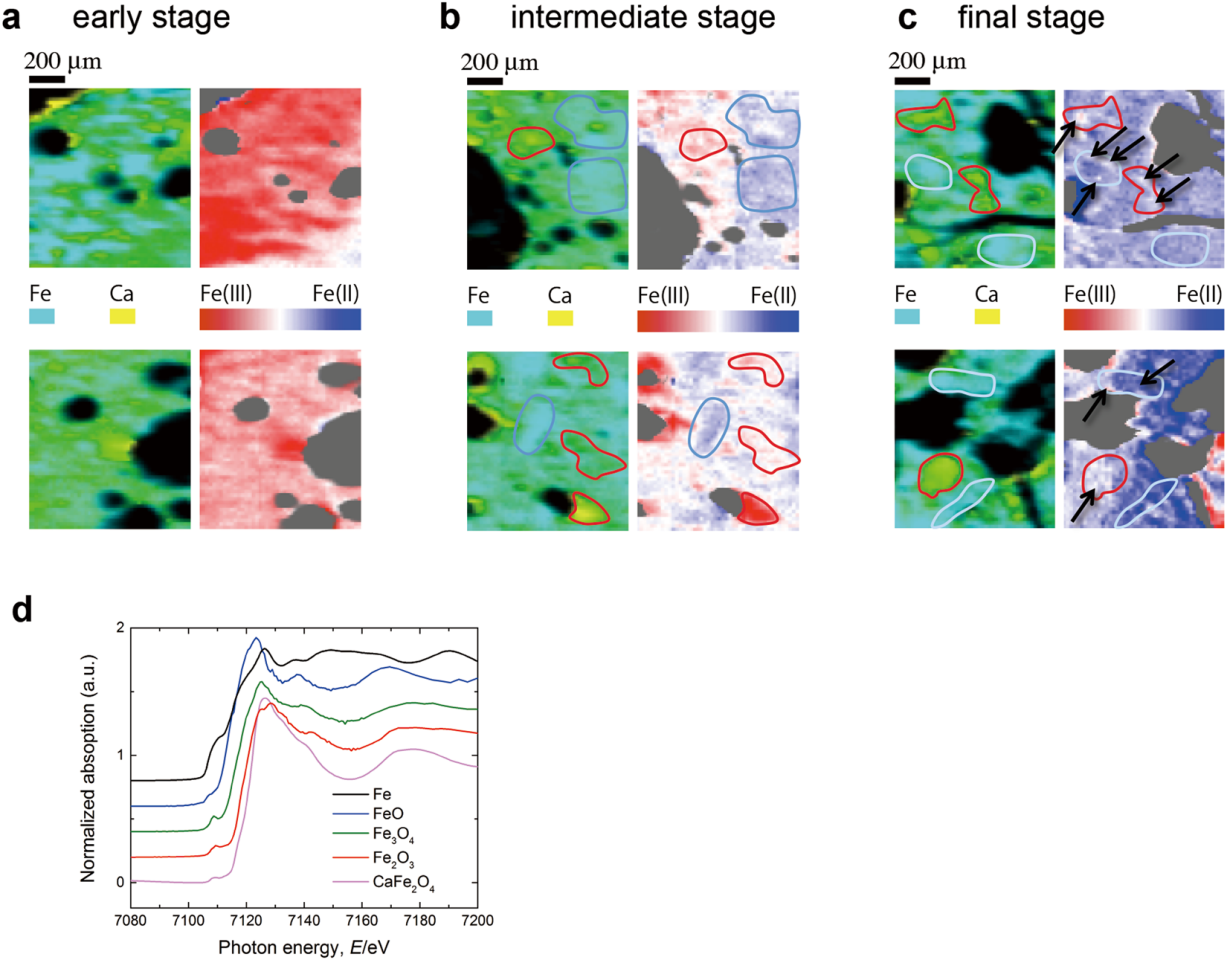
Chemical state mapping of an iron ore sinter. The fraction ratio of Fe(II)/(Fe(II) + Fe(III)) is color-coded: blue for 1 and red for 0. (**a**) Early (sample S-1), (**b**) intermediate (S-2), and (**c**) final (S-3) stages of reduction. The top and bottom rows show the maps for different fields of view of the same specimen for each stage. In each half-panel, the left panel illustrates the elemental distribution of iron (blue) and calcium (yellow), whereas the right panel maps the oxidation states of iron, with the Fe(III) and Fe(II) states coloured red and blue, respectively. The blue lines exemplify regions where the reduction proceeded faster than in other areas, whereas the red lines exemplify regions where the reduction was slower. Even within an area with the same calcium concentration, the reduction progressed heterogeneously, as indicated by the black arrows in Fig. 2c, where the reduction proceeded slower than the rest. (**d**) XANES spectra for standard samples.

The mapping of the valence states of iron oxidation reveals the heterogeneous dynamic evolution of the chemical states from Fe(III) to Fe(II) during the reduction process (Fig. 2). At an early stage of reduction (sample S-1), most areas were populated with Fe(III) states (red), mainly corresponding to α-Fe_2_O_3_ and calcium ferrite phases (Ca_2_(Ca,Fe,Al)_6_(Fe,Al,Si)_6_O_20_ (SFCA) and Ca_3_(Ca,Fe)(Fe,Al)_16_O_28_ (SFCA-I)), as determined by X-ray diffraction (see Supplementary Information, Note S1). At an intermediate stage of the reduction process (sample S-2), approximately half of the iron oxide areas were in a reduced chemical state, and the spatial distribution of the changes in the reduced areas was heterogeneous rather than homogeneous, showing that reduction took place at different rates for different areas (heterogeneous reaction) (Fig. 2b). Reduction initially occurred only in regions with a low calcium concentration, wherein the iron chemical state changed from Fe(III) (red) to Fe(III) + Fe(II) (white) or Fe(II) (blue), whereas the iron ions maintained their Fe(III) oxidation state in regions with high calcium concentrations. At the final stage of the reduction (S-3, Fig. 2c), the oxidation state of iron was Fe(II) (blue) in most areas, even though some areas maintained a Fe(III) + Fe(II) state (as shown by the blue lines in Fig. 2c,d). The Fe(II) states correspond to Fe_3_O_4_ and FeO phases, which were formed by the reduction of Fe_2_O_3_ and/or the decomposition of Ca–Fe–O into Ca–O and Fe–O phases that precedes the reduction of calcium ferrites^28,29^.

It was observed that reduction was more likely to proceed in areas with a low calcium concentration, which can be explained by the experimental results obtained from powder specimens of individual phases, in which iron oxides are more easily reduced than calcium ferrites (Fig. 1b)^28,29^. However, it was also shown that reduction progressed heterogeneously within the same calcium ferrite or iron oxide phase, as exemplified by the black arrows in Fig. 2c. This shows that the heterogeneous reduction was not just the result of the different reduction rates of the individual phases. Other factors would have also contributed, such as the porous structure of the calcium ferrite phase, which affects the flow of the reductive gas. Because the gas flow is more effective in porous regions, reduction is accelerated there.

In order to relate the change in chemical state with crack formation, the changes in the bonding regions during reduction were investigated by X-CT (see Methods, Section 2). As reduction progresses, the iron chemical state changed from Fe(III) to Fe(III) + Fe(II) and finally to Fe(II). This change corresponds to the formation of the Fe_3_O_4_ and FeO phases mentioned above, and their formation could be clearly observed by X-CT because of the large difference in the densities of calcium ferrites and iron oxides (see Supplementary Information, Note S3). In a data set, a 2 × 2 × 10 mm^3^ specimen was divided in voxels of 4 × 4 × 4 μm^3^, and each voxel was identified as (a) initial pores, (b) microcracks formed during reduction, (c) calcium ferrite phases, and (d) iron oxide phases formed during reduction. It was clearly observed that small iron oxide regions are formed in the bonding region of the calcium ferrite matrix as reduction progresses and that the number of cracks increases. However, the change in the microstructure (i.e. the heterogeneity of the phase mapping) is very complicated; thus, we cannot determine how the progress of heterogeneous reduction causes crack formation nor empirically identify trigger sites.

### Persistent homology analysis of image data to identify trigger sites

The correlation between the type and microstructure of the phases involved in the reduction of iron ore sinters and the formation of cracks was investigated using persistent homology^23,26,27^. Having determined the most representative topological features characterizing the reduction process by a persistence diagram (PD), the trigger sites for crack formation were identified using least absolute shrinkage and selection operator (LASSO) regression techniques to determine the correlation between the evolution in the topological features in the calcium ferrite and iron oxide phases comprising the iron ore sinters and the microcracks formed during reduction (see Methods, Section 3).

The first and most important step in our approach involves transforming each image into a PD. We analysed the persistent topological features of the image datasets of calcium ferrites and iron oxides for all slices of specimens S-1, S-2, and S-3 having dimensions of 2 mm × 2 mm × 4.0 μm^3^ (thickness) (in volumes of 2 mm × 2 mm × 10 mm). Here, PDs are calculated as follows (see Supplementary Information, Note S4); we enlarge or contract each domain in the image data step-by-step and trace the changes in topological features between each step. Enlargement corresponds to positive steps while contraction corresponds to negative steps. We define the values *b* and *d* as the step of ‘birth’ and ‘death’ of each domain, respectively. That is, the domain appears at the *b*^th^ step and is merged into another domain at the *d*^th^ step. Hence, the value *d* indicates the maximum distance between adjacent domains, whereas *b* indicates the size of the domains. Then, a PD is constructed as a two-dimensional histogram of topological features plotted at those corresponding birth and death values on the (*b*,*d)*-plane. We note that the positive and negative regions on the *d*-axis in the PDs denote ‘island’- and ‘hourglass’-shaped features in the real-space maps, respectively (see Supplementary Information, Note S4).

**Figure 3:**
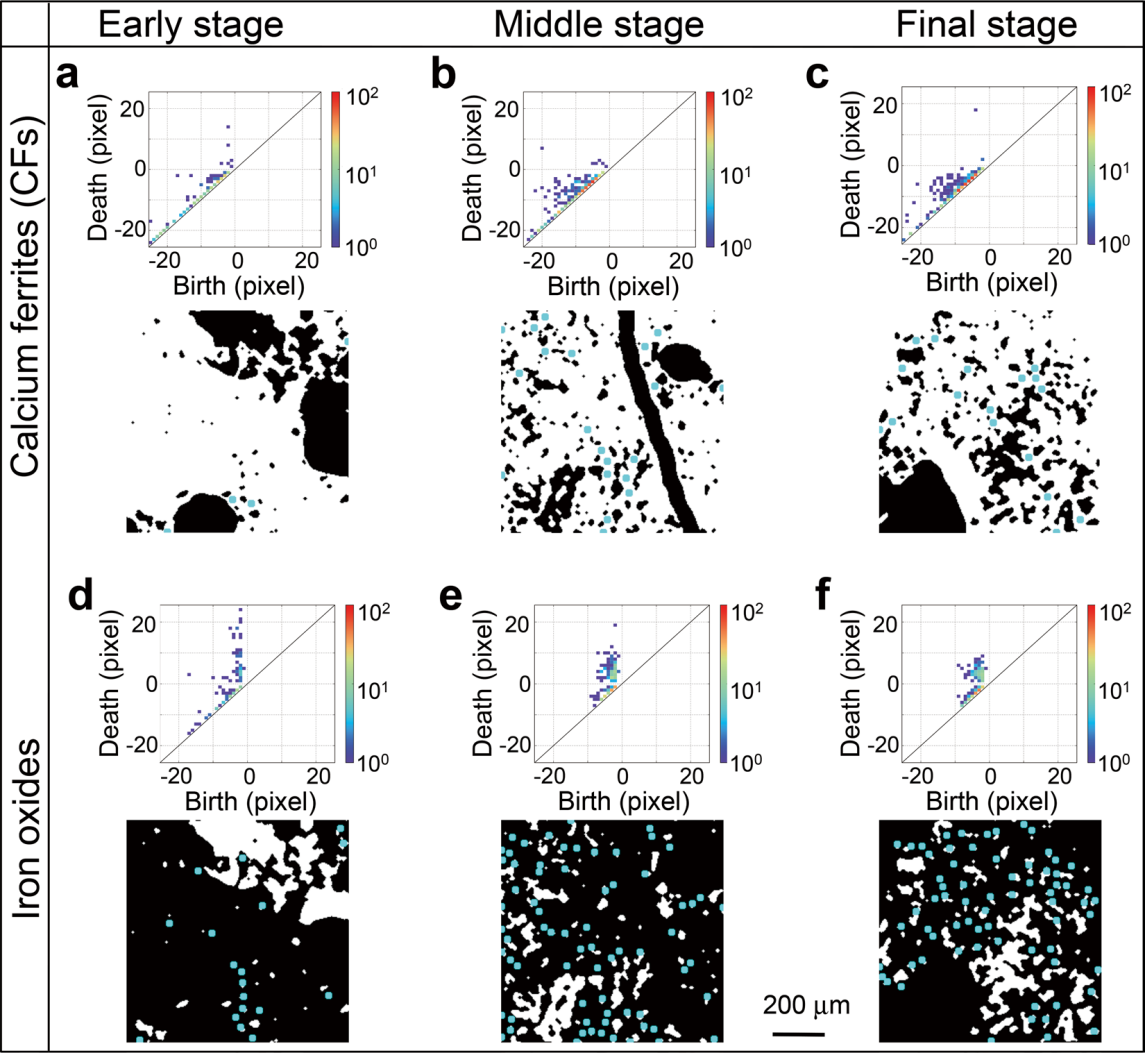
Examples of phase maps (bottom image in each panel) and the corresponding persistence diagrams (upper image in each panel) for (**a**–**c**) calcium ferrites and (**d**–**f**) iron oxides during the (**a**,**d**) early (S-1), (**b**,**e**) intermediate (S-2), and (**c**,**f**) final (S-3) stages of reduction. The blue circles show the centres of the representative shapes in each image (calculated using a PCA analysis) that became predominant as the reduction progressed.

The evolution of the topological features in the phase maps of the calcium ferrite and iron oxide phases were well-captured using PDs. Figure 3 shows examples of mapping images of calcium ferrites and iron oxides with their corresponding PDs. The PDs of calcium ferrites in Fig. 3 show a shift of a highly populated region from the region near (*b*, *d*) = (0, 0) to a region of negative *b* and *d* as the reduction progresses from the early stage to middle and final stages, suggesting that large matrices of calcium ferrites change into many narrow hourglass-shaped grains. In contrast, the PDs of iron oxides show a highly populated region scattered around (0, *d*) (*d* > 0) in the early stage and that the scattered range of *d* becomes smaller as reduction progresses, suggesting that large islands of iron oxide domains change into small ones. The PDs in Fig. 3 suggest that the heterogeneous progress of the reduction process resulted in the gradual formation of iron oxide islands and calcium ferrite hourglass shapes as reduction progressed.

We, then applied machine learning to identify statistical signatures between crack formations and topological features extracted by persistent homology for phase mapping of calcium ferrites and iron oxides. The PDs were converted into a vector representation termed persistence image (PI) and analysed using principal component analysis (PCA) (see Supplementary Information, Note S5). The results of the PCA clearly showed that the S-1 (early stage) dataset is separated from the intermediate and final datasets, S-2 and S-3. The main difference between the early- (S-1) and later- (S-2, S-3) stage data was the number of small iron oxide island shapes and calcium ferrite hourglass shapes, which increased from the early stages to the later stages but did not change very much between the intermediate and final stages (see Supplementary Information, Note S5).

The LASSO was applied to the vector representation of the PDs. Since each element of the vector corresponds to a grid in the histogram, we can identify a subset of grids in the histogram which has the largest impact on the dependent variable: the area of the microcracks; this subset corresponds to the dominant birth–death pairs that have the closest correlation with the areas containing microcracks (see Supplementary Information, Note S5 and S6). To demonstrate the simplicity and validity of our approach, we utilized the macroscopic mechanical property for each slice in the volume data using the sum of crack volume. Alternatively, we might directly measure the mechanical properties of specimens in sufficient detail to conduct machine learning and determine trigger sites using our new approach.

**Figure 4:**
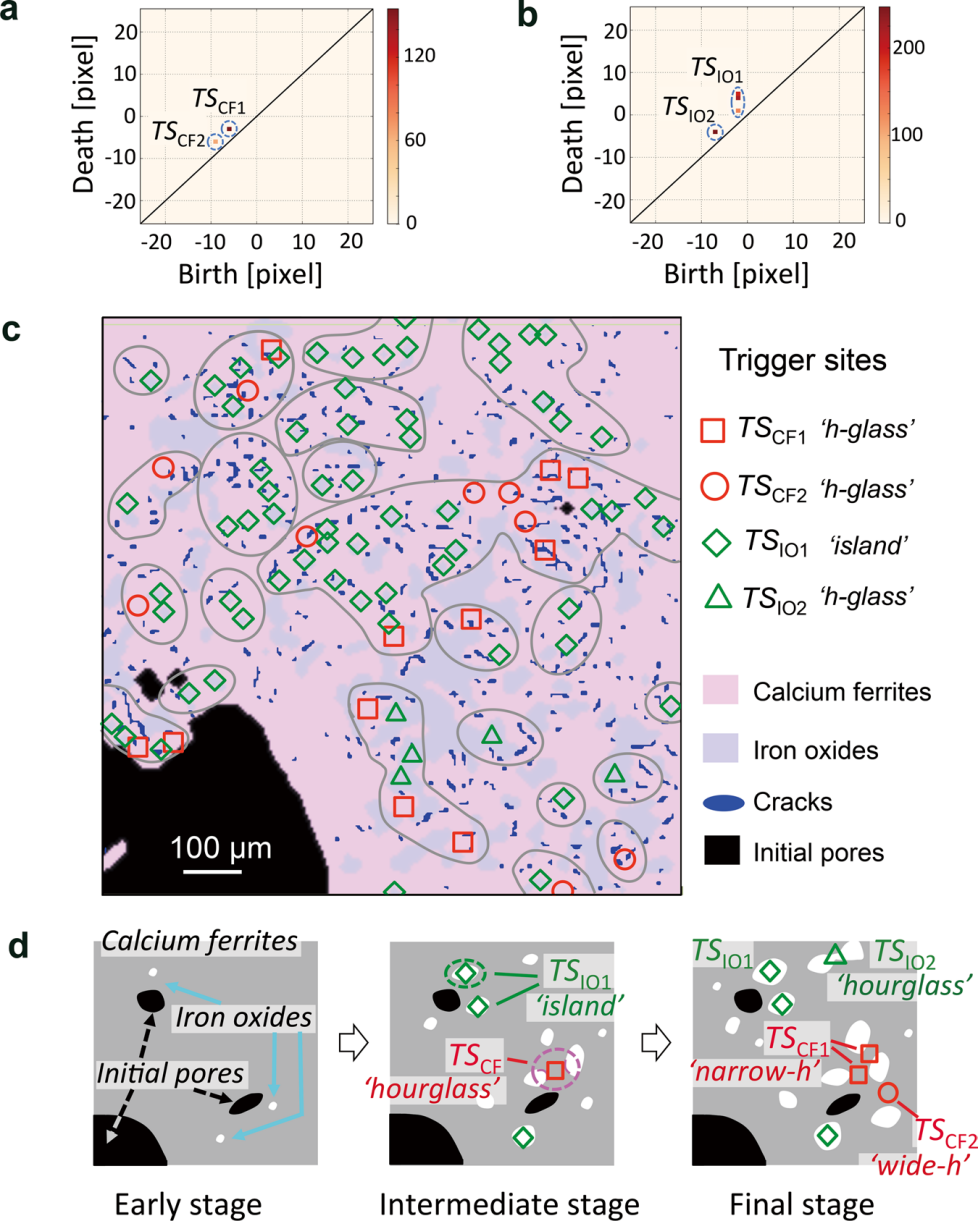
Results of the LASSO analysis and identified trigger sites. (**a**,**b**) Results of the LASSO analysis for the calcium ferrites and iron oxides, respectively. (**c**) Trigger sites in the calcium ferrites (*TS*_CF1_ and *TS*_CF2_) and iron oxides (*TS*_IO1_ and *TS*_IO2_), corresponding to the topological features in Fig. 4a and b, respectively, which are highly correlated with crack formation. The pink and blue matrix-regions represent the calcium ferrite and iron oxides phases, respectively. The black and blue ones show the initial pores and the cracks formed during reduction. (**d**) Schematic of the reduction process and the trigger sites employed: ‘hourglass’-shaped features of calcium ferrites (*TS*_CF_) and ‘island’- and ‘hourglass’-shaped features of iron oxides (*TS*_IO_).

Figure 4a,b show 2D histograms corresponding to the learned vectors obtained from the LASSO analysis on the images of the calcium ferrite and iron oxide phases and of the microcrack regions. The histograms highlight specific regions characterized by the high values of the learned vectors (marked as *TS: trigger sites* in the figures). Four types of dominant birth–death pairs were identified, two for calcium ferrites (*TS*_CF1_ and *TS*_CF2_) and two for iron oxides (*TS*_IO1_ and *TS*_IO2_), each of which are highly correlated with crack formation. These highly correlated components were transformed back into the corresponding real-space locations in specimens (Fig. 4c). In this way, we successfully identified the locations in real space that correspond to the highly correlated birth–death pairs in Fig. 4a,b, i.e. the trigger sites that induced crack formation during the heterogeneous reduction. It should be stated again that the key to the identification of trigger sites is the high correlation between the main topological features corresponding to the calcium ferrite and iron oxide phases and the microcracks.

Herein, we briefly explain the advantages of our method compared to other strategies. First, we can use other simpler descriptors of images such as mean, variance, and higher order moments of pixel values, Gray-Level Co-Occurrence Matrix (GLCM) statistics, connected component counting, and others^14,15^. However, the selection of appropriate descriptors requires prior knowledge for selecting appropriate one, a disadvantage that has been overcome using our data-driven method. Another strategy for image analysis involves directly applying standard machine learning methods, such as bag-of-keypoints with kernel support vector machine and deep neural networks^11–13^. However, these methods naturally create a black box, which prevents inverse analysis of the original real-space. In contrast, our method provides a pipeline for statistical inverse analysis, which provides satisfactory insights into the reaction mechanism; one of the significant advantages to others. Other groups have reported the successful combination of machine leaning with PDs in materials science^21,22,25^. These works construct descriptors for machine leaning from persistent homology, which works well if given prior knowledge or intuition about the data set as mentioned above, which is unfortunately unavailable for our application. Several performance comparisons showing the advantages of our method are given in Supplementary Information, Note S5.

It should be noted that the macroscopic mechanical properties of a sinter are not simply the sum of mechanical properties of local areas (in the order of tenths of μm^2^). This case differs from that of functional properties such as electronic or heat conductivity. Thus, simple comparisons between the locations of microcracks with the microstructures of calcium ferrites and/or iron oxide phases are insufficient because the locally correlated features do not necessarily correspond to the trigger sites which deteriorate the whole sinter. For example, cracks and certain microscopic features correlating (or coinciding) with cracks do not necessarily lead to the deterioration of macroscopic mechanical properties when they are arranged homogeneously within a specimen. Microstructural features can become trigger sites when they cause a regional or heterogeneously distributed increase in stress in a sinter sample. For this reason, we focus on microstructural features that correlate with the overall microscopic mechanical properties by using a homological approach and machine learning rather than a direct comparison between the distribution of cracks and phases.

## Discussion

Four types of trigger sites were identified by persistent homology in data-driven way without any prior knowledge about the materials and the processes involved (Fig. 4c). The trigger sites labelled *TS*_CF1_ and *TS*_CF2_, located in negative death regions in Fig. 4a, were found in the hourglass-shaped features of the calcium ferrites with a narrow width of 8–14 μm (*TS*_CF1_ has a smaller width than *TS*_CF2_) (Fig. 4d). Another set of trigger sites, *TS*_IO1_ and *TS*_IO2_, was found in the island- and hourglass-shaped iron oxide features, respectively (note: *TS*_IO1_ and *TS*_IO2_ are located in the positive and negative death regions in Fig. 4b, respectively); these sites had approximate sizes of 2 and 8 μm, respectively (Fig. 4d).

The determined trigger sites are reasonable in terms of materials-scientific knowledge. The trigger sites populating the hourglass features of the calcium ferrites (*TS*_CF1_ and *TS*_CF2_), and the island (*TS*_IO1_) and hourglass (*TS*_IO2_) features of the iron oxides, were presumably formed as a result of the decomposition of Ca–Fe–O into the Ca–O and Fe–O phases before the reduction of the calcium ferrites, as found in a period from 0 to 3 min in CaFe_2_O_4_ (Fig. 1b)^28,29^, leading to the formation of complex calcium ferrites and iron oxide microstructures. The correlation between these topological features and the formation of cracks is certainly reasonable because iron reduction in the calcium ferrite and iron oxide phases is accompanied by a large volume decrease^6^ and leads to an increase in the local stress (and then to crack formation) due to the different reduction rates of the calcium ferrites and iron oxides^28,29^.

The role of the trigger sites in the crack formation during reduction can be explained as follows (Fig. 4d). In the early stages of the reaction, only a few trigger sites were found because only a slight increase in the stress is expected at this time. At intermediate stages, large areas of iron oxides and calcium ferrites were separated into complex microstructures as a result of the progress of reduction. Larger numbers of trigger sites were found in the fine microstructures and not limited to the areas near the pores; the trigger sites occupying the iron oxide islands (*TS*_IO1_) and the hourglass-shaped regions of the calcium ferrites (*TS*_CF1_ and *TS*_CF2_) remained spatially separated from each other. In the final stage of the reduction process, the trigger sites spread across the entire specimen, and the two types of trigger sites overlapped, which makes small and isolated cracks aggregate and causes large macroscopic cracks resulting in fracture of the specimen.

It has been clearly demonstrated that the present method based on persistent homology and statistical analysis can identify the trigger sites of the reduction of iron ore sinters using only the image data of phase mapping and crack formation. The identification of the specific locations of crack formation during the reduction process has never been available using conventional and empirical approaches, although the type of microstructure has been believed to play important roles^3–5^. The identification of trigger sites can be used to quantitatively predict the fracture toughness or lifetime of iron ore sinters. For example, we may predict the fracture toughness of an iron ore sinter on the basis of the size and number distributions of hourglass-shaped calcium ferrites and island-shaped iron oxides. Furthermore, on the basis of our findings, the mechanical properties of iron ore sinters can be improved by changing the initial microstructures of calcium ferrites into finer ones, which may prevent an increase in the local stress from aggregating into a macroscopic stress. A change in microstructure may be achieved by controlling the heating pattern of sintering^30^ or the chemical compositions of calcium ferrites, such as silicon and/or aluminium^31,32^. This is expected to suppress the degradation of sinters during reduction, resulting in an increase in the process efficiency and reductions in energy and natural resources (iron ores).

This work showed the power of the proposed method to analyse a heterogeneous reaction without requiring specific data about the reaction mechanism involved. In the process, only image data depicting the heterogeneity of a material, such as a map of the chemical states and coexisting phases, and their relationship to a macroscopic property are required, which are analysed by persistent homology and machine learning. The proposed method can be applied to find trigger sites, as far as the features of heterogeneity governing the macroscopic properties. For studying the correlation among three or more co-existing phases, a naive approach is to apply pairwise analysis of two-phase correlations, although it may make the analysis complicated.

The proposed method can be easily extended to various types of datasets with heterogeneity, even in the case of large and multi-dimensional datasets produced using current analytical methods such as *in situ* X-CT, XRM, and other techniques of mapping heterogeneity. It also has the potential to be a powerful tool for predicting trigger sites in heterogeneous processes without any specific information about the reactions involved. For example, when macroscopic properties such as the electrical or thermal conductivity are determined by the heterogeneity of coexisting phases, this method could provide most important topological features or trigger sites without information regarding the mechanism such as the interactions among the domains of coexisting phases. Hence, the proposed method could be applied to gain further insights into different systems for which no specific knowledge of the reaction mechanism is available.

## Methods

### Preparation of iron ore sinters

Sinter specimens were prepared by liquid sintering from iron ore and limestone. Natural iron ore particles (mainly Fe_2_O_3_, a few millimetres in diameter) were mixed with limestone flux (CaO, 7.4 mass%) and coke breeze (C, 4.0 mass%) and heated via the combustion of coke breeze, increasing the temperature from 1450 to 1600 K (above the eutectic temperature of CaO–Fe_2_O_3_, 1478 K). Iron ore sinter samples were prepared using natural iron ore and calcite minerals including silica and alumina as impurities. The chemical compositions of specimens are, Fe:30.9, O:59.2, Ca:5.6, Si:3.1, Al:1.2 by at%. Then, the specimens were heated up to 1473 K in a CO/CO_2_ (from 50:50 to 80:20, 10^−1^ MPa) reductive gas atmosphere for reduction, simulating the iron-making process. Different degrees of reduction were obtained by quenching the specimen at different temperatures and CO partial pressures: 873 K (early stage, sample S-1) to 1173 K (intermediate stage, sample S-2) and P1473 K (final stage, sample S-3). Specimens obtained with increasing reduction temperatures and CO partial pressures have a higher degree of reduction, which was confirmed by X-ray diffraction (Supplementary Information, Note S1 and Table S1).

### Chemical state/phase mapping and X-CT measurements

Chemical state mapping^29^ was carried out on the basis of X-ray absorption measurements at the synchrotron undulator beamline BL-15A1^33,34^ of the Photon Factory, IMSS, KEK in Japan. A sinter was embedded in a resin and sliced into specimens with a thickness of 20–30 μm. XANES spectra were measured using ion chambers located before and after the specimen to measure the incident and transmitted intensities (see Supplementary Information, Fig. S2). A XANES spectrum was expressed as a linear combination of the Fe^II^O and Fe^III^_2_O_3_ components, and the ratio between the amounts of the two phases was determined by a least-squares fitting^29^. X-ray fluorescence spectra were also measured using a silicon drift detector (SDD) in order to determine the iron and calcium contents. The typical time required for chemical mapping using 30 photon energies was a few hours for a 1 mm square with an ‘on the fly’ mode.

The crack formation in and phase mapping of larger volumes (2 × 2 × 10 mm^3^) were investigated using X-CT measurements performed with an in-house X-ray source. For the persistent homology analysis, the X-CT datasets of the reduced sinter were deconvoluted into (a) the initial pores, (b) the microcracks formed during reduction, (c) calcium ferrite phases, and (d) iron oxide phases (see Supplementary Information, Note S3).

### Persistent homology analysis

The analysis involves (1) transforming each image into a PD and then (2) into a vector, (3) feeding the vectors together with the measured crack areas into the LASSO, (4) identifying the dominant birth–death pairs, and finally (5) mapping them back into the original image to reveal the real-space topological (persistent) features. (see Supplementary Information, Note S4 for (1) and Note S5 and S6 for (2), (3) and (4))

(1) PDs were computed from each slice with dimensions of 2 mm × 2 mm × 4.0 μm^3^ of a calcium ferrite and iron oxide phase image obtained by X-CT. (2) PDs were converted into a finite-dimensional vector representation called the persistence image (PI) in order to analyse them using machine learning. The PIs were then analysed using PCA, which found the lowest-dimensional representation of the vectors in the PIs. (3) The LASSO tool was used to detect the dominant birth–death pairs with the closest correlation with the areas of microcracks. (4) Four types of dominant birth–death pairs were identified, two for calcium ferrites and two for iron oxides. (5) They were mapped back into the original image, identifying the trigger sites in the reduction process.

### Data availability

The authors will make available, upon request, typical data described in this work. It is understood that the data provided will not be for commercial use.

## Electronic supplementary material

Supplementary Information
