# Supplementary material for: Non-empirical identification of trigger sites in heterogeneous processes using persistent homology
Source: Sci Rep. 2018 Feb 23;8:3553. doi: 10.1038/s41598-018-21867-z (PMC5824834; doi:10.1038/s41598-018-21867-z)
Supplement: Supplementary file 1 — Supplementary Information [file 41598_2018_21867_MOESM1_ESM.pdf]

Supplementary Information

Non-empirical identification of trigger sites in heterogeneous processes using persistent homology

M. Kimura*[1,2], I. Obayashi[3], Y. Takeichi[1,2], R. Murao[4], Y. Hiraoka[3,5,6]

[1]Photon Factory, Institute of Materials Structure Science, High Energy Accelerator Research Organization, Tsukuba, Ibaraki 305-0801, Japan

[2]Department of Materials Structure Science, School of High Energy Accelerator Science, SOKENDAI (The Graduate University for Advanced Studies), Tsukuba, Ibaraki 305-0801, Japan

[3]Advanced Institute for Materials Research (AIMR), Tohoku University, 2-1-1 Katahira, Aoba-ku, Sendai 980-8577, Japan

[4]Advanced Technology Research Laboratories, Nippon Steel & Sumitomo Metal Co., Futtsu, Chiba 293-8511, Japan

[5]Center for Materials research by Information Integration (CMI2), Research and Services Division of Materials Data and Integrated System (MaDIS), National Institute for Materials Science (NIMS), Tsukuba, Ibaraki 305-0047, Japan

[6]Center for Advanced Intelligence Project, RIKEN, Tokyo 103-0027, Japan

Note S1. XRD analysis of specimens

We used X-ray diffraction (XRD) with a Cu K_α X-ray source to identify the coexisting phases in the pulverized specimen and their crystal structures. The XRD patterns and volume fractions of the detected phases are shown in Fig. S1 and Table S1.

As shown by chemical state map, the iron chemical state changed from Fe(III) to Fe(III) + Fe(II) and finally to Fe(II) during reduction. The results in Table S1 clearly demonstrate that specimens obtained at increasing reduction times have higher mass fractions of the Fe_3O_4 and FeO phases, which contain iron atoms in the chemical states of Fe(III) + Fe(II) and Fe(II), respectively. The Fe_3O_4 and FeO phases were formed by the reduction of Fe_2O_3 and/or the decomposition of Ca–Fe–O into the Ca–O and Fe–O phases, which precedes the reduction of calcium ferrites.

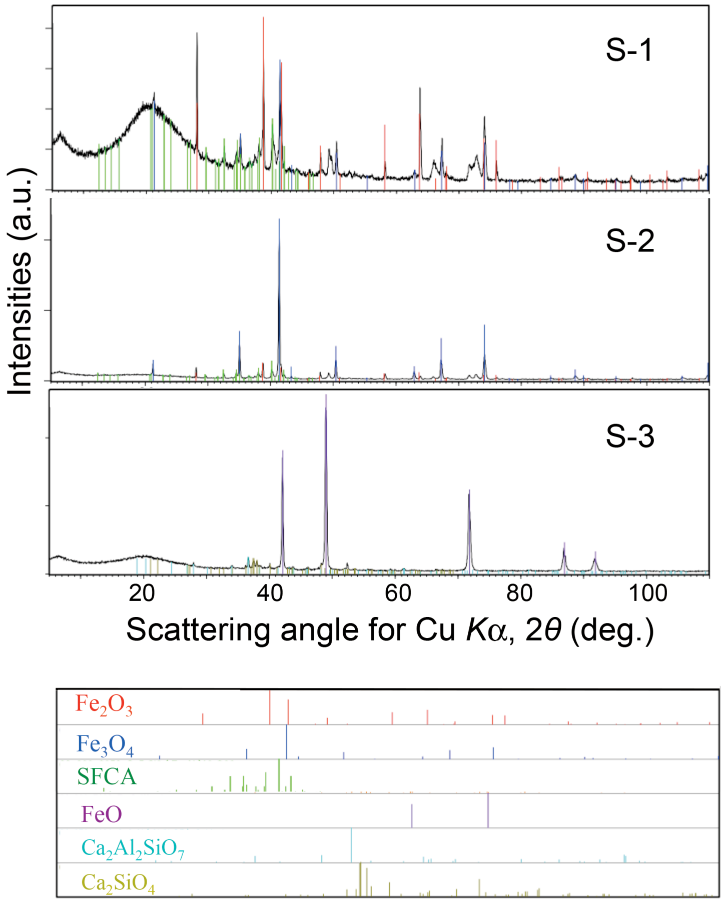

Fig. S1. XRD patterns of the specimens (top) and references (bottom).

Table S1. Results of the XRD analysis. (**a**) Mass percentages of the detected phases in the specimens determined by the Rietveld analysis and (**b**) crystallographic parameters of the calcium ferrites (SFCA [1] and SFCA-I [2]) used for initial structural models of the Rietveld analysis.

a

	α-Fe_2O_3	Fe_3O_4	FeO	Calcium ferrites (SFCA + SFCA-I)	Ca_2SiO_4	$Ca_2Al_2SiO_7$
S-1	44	19	1	32	4	n.d.
S-2	15	70	3	8	4	n.d.
S-3	1	4	78	5	10	2

b

Phase	$Ca_{2.8}Fe_{8.7}Al_{1.2}Si_{0.8}O_{20}$ (SFCA)	$Ca_{3.18}Fe_{15.48}Al_{1.34}O_{28}$ (SFCA-I)
Crystal structure	Space Group: $P\overline{1}$, a=0.90610nm, b=1.00200nm, c=1.09200nm, α=60.300º, β=73.680º, γ =65.810º, V=0.781762nm^3	Space Group: $P\overline{1}$, a=1.03922nm, b=1.05945nm, c=1.17452nm, α=94.308º, β=111.293º, γ =109.647º, V=1.105700nm^3

Note S2. Chemical state mapping

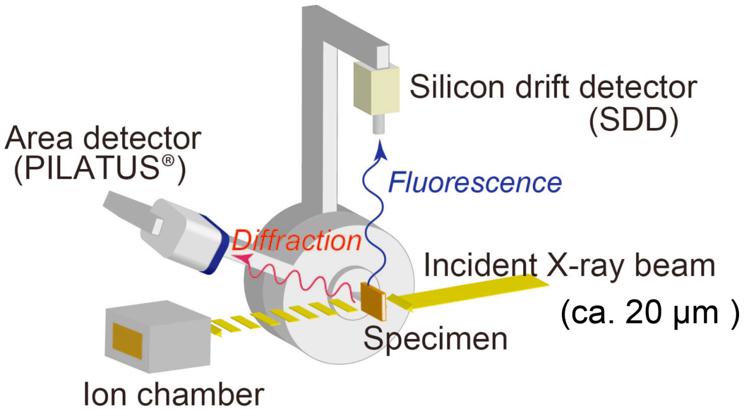

Fig. S2. Chemical state mapping experiments[3]. Schematic of the experimental setup for chemical state mapping at the BL-15A1 beamline[4,5] of the PF, KEK (Japan)

Note S3. X-CT data and image processing

Crack formation was investigated using X-ray computer tomography (X-CT) with an in-house X-ray source. A cuboidal specimen with dimensions of $2 \times 2 \times 10$ mm^3 was cut from an iron ore sinter. A CCD camera with a scintillator was used as the detector. A transmission image was measured in 1° or 2° steps for a 360° rotation using a white X-ray source generated by a tungsten target with a tube voltage of 70 keV. The minimum spatial resolution was as small as 0.7 μm. The leftmost column in Fig. S3 shows images of slices extracted from the three-dimensional (3D) X-CT dataset of the reduced sinter that have been reconstructed from the observed data. Panels (a)–(d) show the deconvolution of the microstructure into images representing (a) the initial pores, (b) the microcracks formed during reduction, (c) calcium ferrite phases, and (d) iron oxide phases. These components were extracted for three regions (pores/cracks, calcium ferrites, and iron oxides) by setting threshold values for the image contrast corresponding to the densities and considering the contrast differences observed at the boundaries. Each image of a slice with a thickness of 4.0 μm was processed with a spatial resolution of 4.0 μm. In other words, the $2 \times 2 \times 10$ mm^3 specimen was divided in voxels of $4 \times 4 \times 4$ μm^3, and each voxel was assigned to (a) an initial pore, (b) a microcrack, (c) a calcium ferrite phase, or (d) an iron oxide phase. This dataset was further analysed using persistent homology.

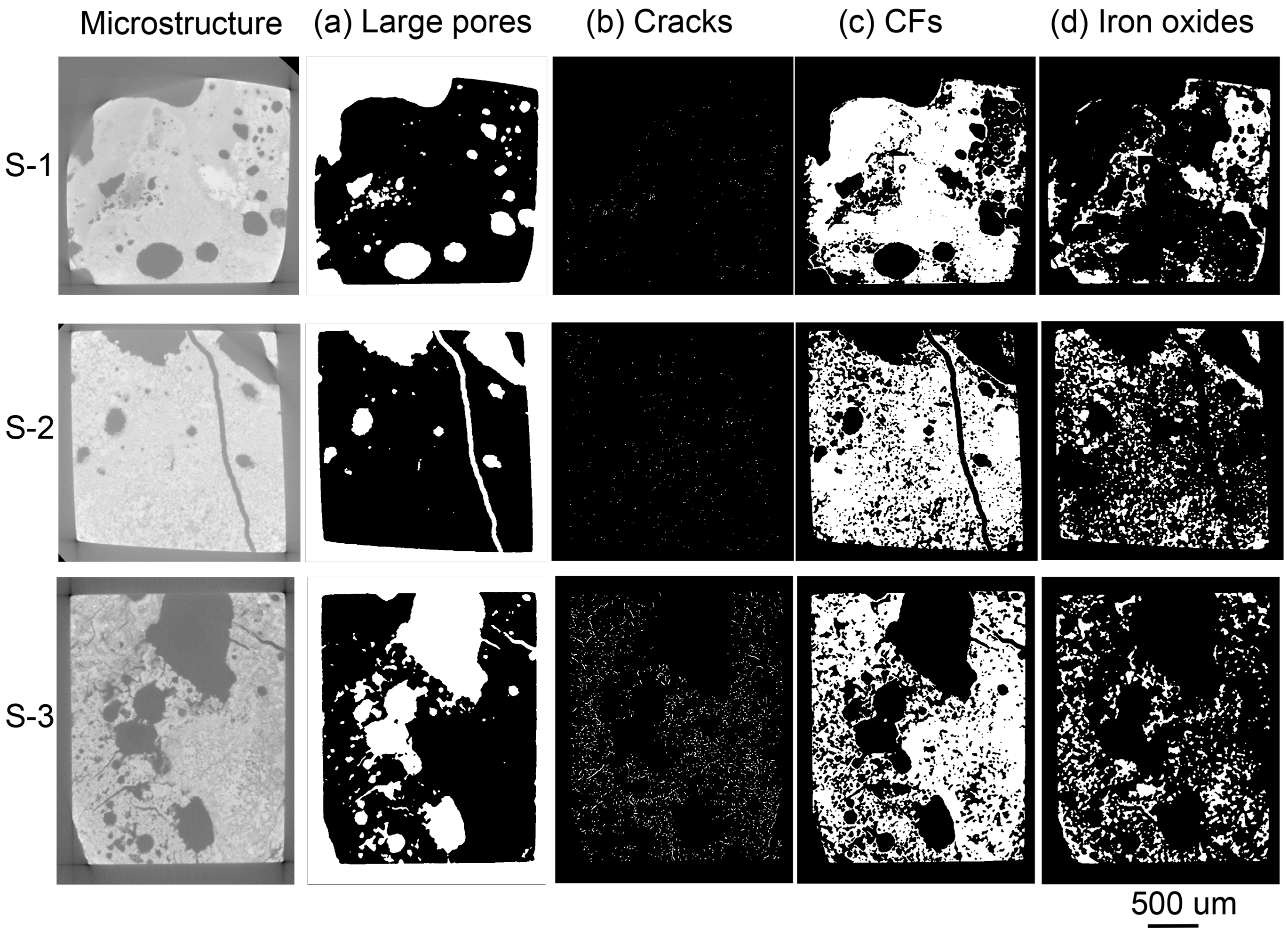

Fig. S3. Microstructures obtained by X-CT and subsequent image processing. The left column displays images of slices extracted from the three-dimensional X-CT dataset of the reduced sinter, corresponding to the early (S-1), intermediate (S-2), and final (S-3) stages of reduction. (a–d): Deconvolutions of the microstructures into (a) the initial pores, (b) the microcracks, (c) calcium ferrites, and (d) iron oxides.

Note S4. Persistence diagrams from image data

Persistence diagrams (PDs)[6-9] are computed from finite points in space or binary images and have been used in the structural analysis of amorphous solids[8] and granular media[9]. Here, in order to quantify the topological features of holes in the phase-mapping data sets obtained by X-CT, we computed the 0-th PDs of those data sets, where we consider iron oxides as 'holes' in the matrix of calcium ferrite. It should be noted that we could characterise topological features in the opposite manner, i.e., calcium ferrites as 'holes'. We performed the calculation in both manners and confirmed their consistency.

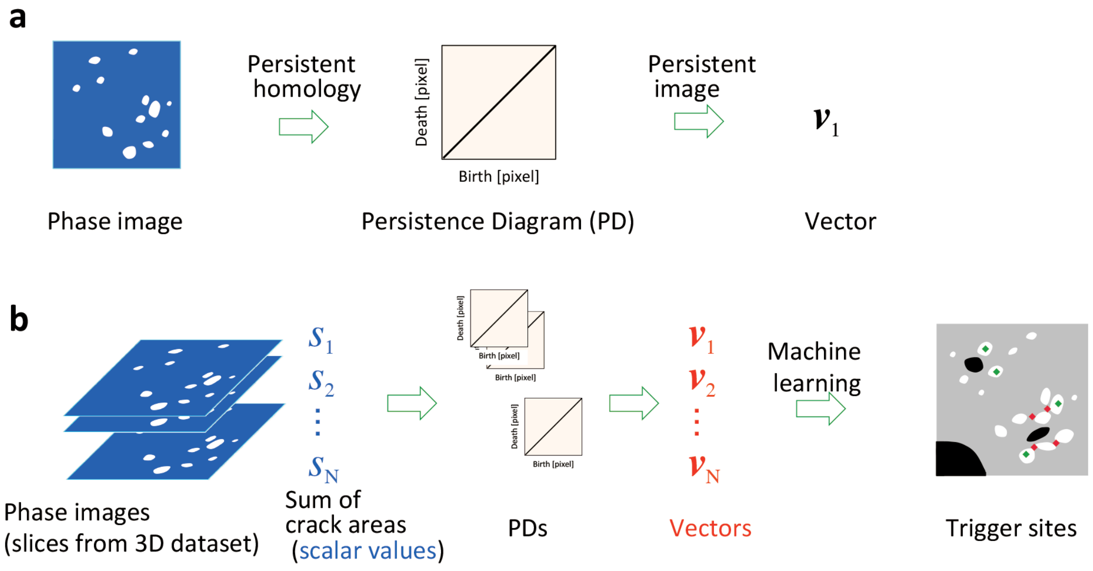

Fig. S4. Outline of the analysis based on persistence diagrams. (**a**) Phase image data obtained from all slices of a 3D dataset are first transformed into PDs and then into vectors. (**b**) The resulting vectors are analysed using machine learning.

Figure S4 outlines the computational framework employed. The analysis involves (1) transforming each image sliced from the 3D data sets into a PD and then (2) into a vector and (3) feeding the vectors together with the measured crack areas into the least absolute shrinkage and selection operator (LASSO), (4) identifying the key birth–death pairs, and finally, and finally (5) mapping them back into the original image to reveal the real-space persistent topological features identifying the trigger sites in the reduction process.

In this note, we discuss how to compute the 0-th PD of an image using the example shown in Fig. S5. Fig. S5a shows the input binary image. We focus on the white pixel island-type shapes (complementary, grey holes). The 0-th PD of the image is calculated to

be {(-3,∞), (-2, -1), (-2, 3)} (Fig. S5b) as follows. First, each pixel in the image is numbered as in Fig. S5c, and these numbers are used to compute the diagram. The white and grey pixels are numbered negatively and positively, respectively. The rule for assigning these numbers, known as the Manhattan distance, is given as follows:

- All grey pixels next to the white pixels are numbered as 1.
- All not-yet-numbered grey pixels next to '1' pixels are numbered as 2.
- In the same way, all not-yet-numbered grey pixels next to '*k*' pixels are numbered as *k + 1* (*k* = 2, 3, 4, …).
- All white pixels next to the grey pixels are numbered as -1.
- In the same way, white pixels are numbered as -2, -3, etc.

Using these assignments, we can enlarge or contract each white region ('island' domains) of the binary image by changing a threshold value *M*. Namely, we define the white regions as the union of all pixels whose assigned number is less than or equal to *M* (Fig. S5d). This enlargement/contraction process results in the appearance ('birth') and the disappearance ('death') of some islands as *M* changes. The initial image (Fig. S5a) corresponds to step = 0 (*M* = -1). Figure S5d shows the evolution of island domains when changing *M* from -3 to 3 (step -2 to 3). An island is born at *M* = -3, as indicated in blue. Increasing *M* to -2, the island becomes larger, and two other islands are born. At *M* = -1, the islands born at *M* = -3 as indicated in blue and red, and -2 merge into one island, which further merges at *M*=3 to the other island born at *M* = -2. Thus, the topological features of the input image (Fig. S5a) are summarized as follows: two islands are born at *M* = -2, die at *M* = -1 and 3, respectively, and the island at *M* = -3 never dies. We encode these birth and death events as pairs of those threshold values *M*, and the 0-th PD is defined by the collection of these birth-death pairs $\{(-3,\infty),(-2,-1),(-2,3)\}$ (Fig. S5b). Hence it can be expressed as a 2-dimensional histogram on the birth-death plane. Following the same process, the PD of the real phase mapping data has the general form $\{(b_k,d_k)\}_{k=1}^{M} \cup \{(b_{M+1},\infty)\}$, where each set of (b_k,d_k) and (b_{M+1},∞) represents the birth–death pair.

Here, we summarize some of the important topological features encoded in the birth-death pairs.

● Each birth–death pair with a positive death value corresponds to an island in the original input image, and the magnitude of the birth value of the pair gives the size (radius) of the island. The birth–death pairs $(-3,\infty)$ and $(-2,3)$ in the above example correspond to the two white islands in the input image (Fig. S5c).

● Each birth–death pair with a negative death value corresponds to an hourglass shape in the input image. Its width is encoded as the magnitude of the birth value (the half-width of the widest section of the hourglass) and the death value (the half-width of the middle section of the hourglass). The pair $(-2,-1)$ corresponds to such a structure (Fig. 5Sc).

● Birth–death pairs with large differences correspond to persistent topological features. In other words, they show topological features that remain for relatively longer periods during evolution (i.e. the reaction) and are expected to play important roles in initiating trigger sites. It should be noted that the birth position of each island represents the centre of the persistent structure associated with the corresponding birth–death pair (red and blue squares in Fig. S5c).

The 0-th PD calculated in this way captures the topological features characterizing the phase mapping of iron oxides and calcium ferrites in the sinter. In this construction, the birth scale b_k indicates the size of the domains, whereas the death scale d_k indicates the maximum distance between adjacent domains. The PDs in this article were computed using the DIPHA algorithm[10].

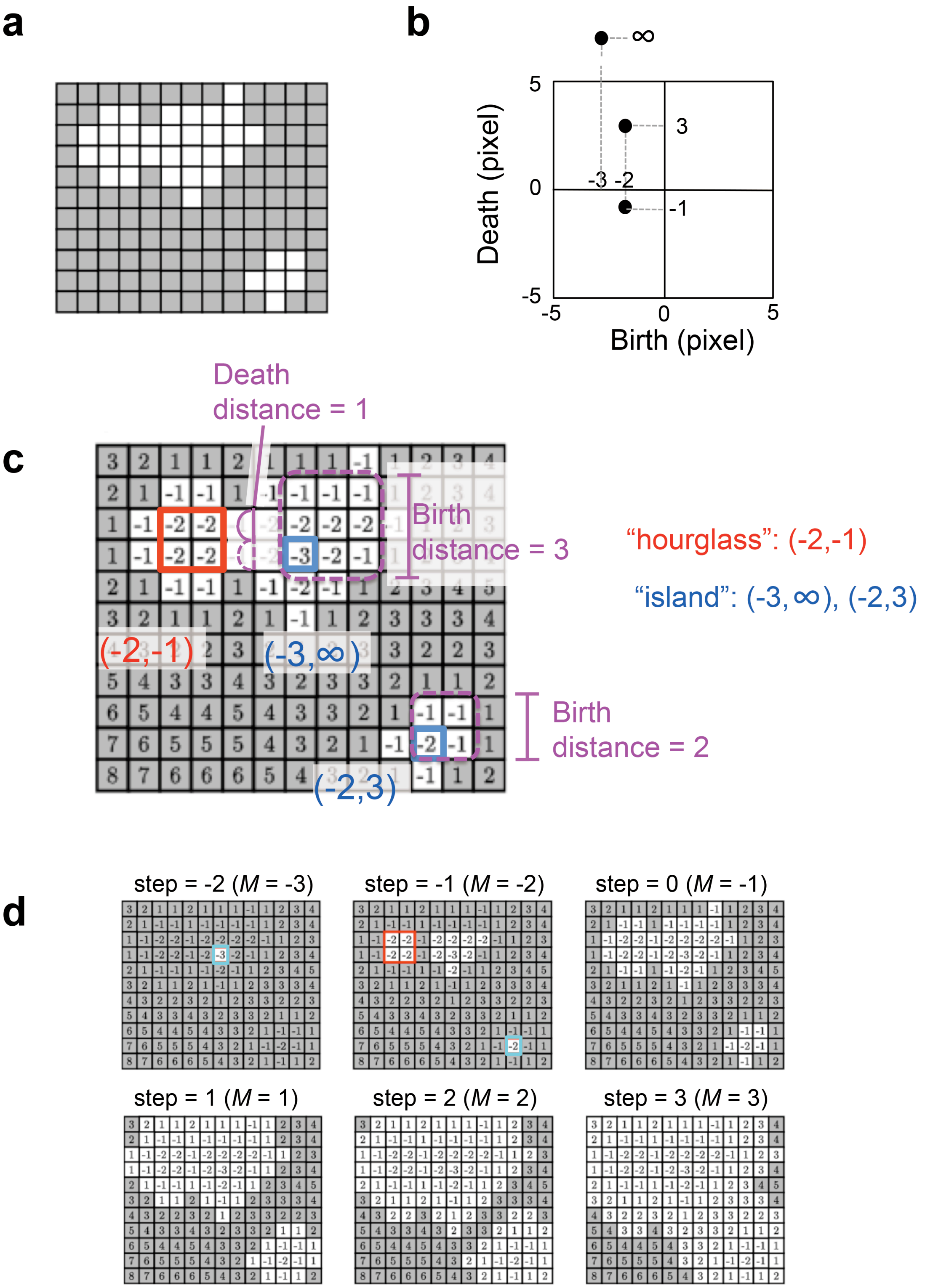

Fig. S5. Computation of PDs. The 0-th PD (**b**) is computed from the input image (**a**), which is numbered as in (**c**) in order to carry out the calculation. As shown in (**d**), the white regions gradually become larger by changing the threshold value M when we define the white regions as the union of pixels whose assigned values are less than or equal to M. This results in the appearance and the disappearance of some islands.

Note S5. Principal component analysis (PCA) of PDs and the identification of trigger sites by linear regression

Among the efforts to map PDs into vector spaces for machine learning tasks, one tool for converting PDs converting to (finite-dimensional) vectors is known as a persistence image (PI). The PI of a given PD is a weighted sum of Gaussian distributions on the birth-death plane and is regarded as a vector in the $L^2(\mathbb{R}^2)$ function space, which is an infinite-dimensional vector space with an inner product. More explicitly, for a PD with $\{(b_k, d_k)\}_{k=1}^{M} \cup \{(b_{M+1}, \infty)\}$, the PI ρ is defined by

$$\rho(x,y) = \sum_{k=1}^{M} w(b_k, d_k)\exp(-\frac{(b_k - x)^2 + (d_k - y)^2}{2\sigma^2}), \tag{S5-1}$$

$$w(b,d) = \arctan(C(d-b)^p), \tag{S5-2}$$

where $C > 0$, $p > 0$, and $\sigma > 0$ are parameters, and $w(b, d)$ is a weight function. Because a birth–death pair with a large difference is expected to be an important topological feature, the weight function is chosen to respect this requirement. In practice, the PI is discretized into a finite-dimensional vector using a histogram on the birth-death plane with a finite mesh. It should be noted that the PI is stable under this transformation with respect to small perturbations in the inputs[11].

Using the vectors of the discretized PIs, we applied machine learning methods to investigate the characteristic topological features of the phase image datasets of calcium ferrites and iron oxides. In particular, we used PCA and linear regression with l^1 regularization (LASSO).

Principal component analysis. PCA is a standard, unsupervised machine learning method[12]. Starting from an input set of vectors in $\mathbb{R}^n$, the PCA finds the lowest-dimensional representation of the vectors. In particular, given an input set of vectors $\{v_1, ..., v_N\}$, the PCA finds the principal components $w_1, ..., w_k \in \mathbb{R}^n$ and their orthogonal projections $p_1, ..., p_k$ so that any vector v_i can be approximated as

$$v_i \approx p_1(v_i)w_1 + \cdots + p_k(v_i)w_k + w_0, \tag{S5-3}$$

where w_0 is the average of $\{v_1, ..., v_N\}$. The k-dimensional vector $(p_1(v_i), ..., p_k(v_i))$ is regarded as the low dimensional representation of v_i. In our setting, the PCA was applied

to the discretized PIs. Furthermore, we reconstructed a histogram from each principal component w_i, which can be regarded as a 'principal PD'. Therefore, the equation (S5-3) requires that each PD was approximated by the weighted sum of the reconstructed histograms with the weights $p_1(v_i),\ldots,p_k(v_i)$. This fact was important for our analysis.

Figure S6 shows the 2D representation of the PCA for (a) calcium ferrites and (b) iron oxides. Each point in this figure corresponds to one PD (and to one image). The figure clearly shows that the S-1 (early stage) dataset is separated from the intermediate and final datasets, S-2 and S-3. The directions w_1-w_2 in Fig. S6(a) and w_1+w_2 in Fig. S6(b) are important for separating sample S-1 from samples S-2 and S-3. By reconstructing PDs from these directions, the PCA shows that the main difference between the early- (S-1) and later- (S-2, S-3) stage data was the number of calcium ferrite hourglass shapes and small iron oxide island shapes, which increased from the early stages to the later stages but did not change very much between the intermediate and final stages.

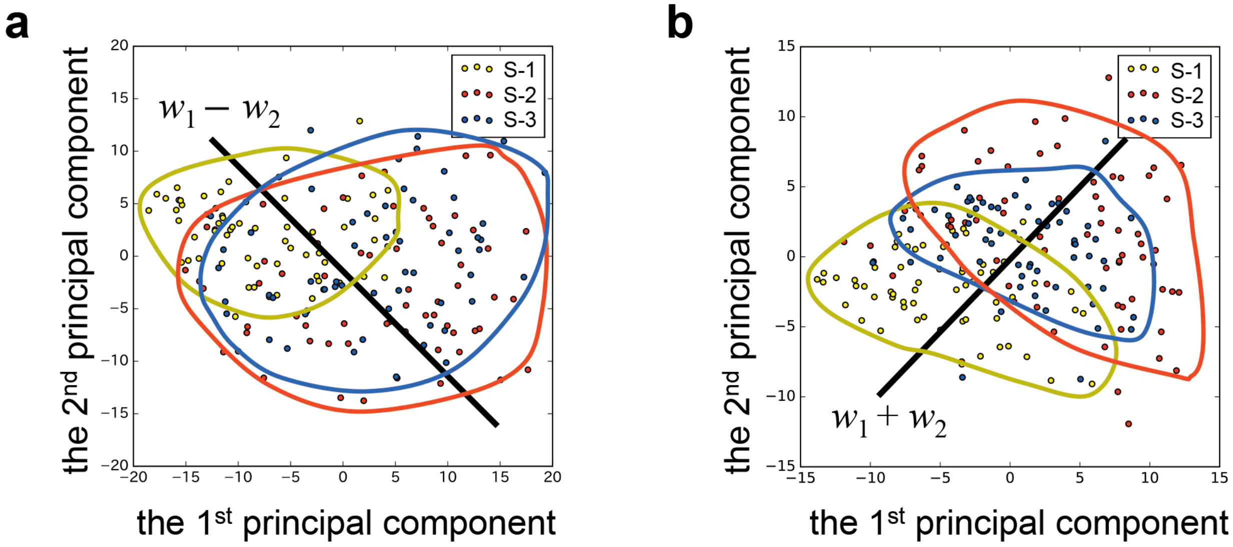

Fig. S6. Results of the principal component analysis of the S-1, S-2, and S-3datasets. (**a**) Calcium ferrites and (**b**) iron oxides.

Comparison with other methods. As described in the main text, the PCA analysis has succeeded in identifying the increase in the number of 'island'- and 'hourglass'-shaped features during the reduction from S-1 to S-3 using minimal prior knowledge, *i.e.* heterogeneous features (or 'shapes') of co-existing iron oxides and calcium ferrites.

Once the types of trigger sites were identified as 'island'- and 'hourglass'-shaped iron oxides and 'hourglass'-shaped calcium ferrites, we can analyse the phase images using a simple image analysis technique. For comparison, Fig. S7 contains histograms showing the number of connected components in the iron oxide images. The number increases from S-1 to S-2 and decreases slightly from S-2 to S-3. This finding is consistent with PCA.

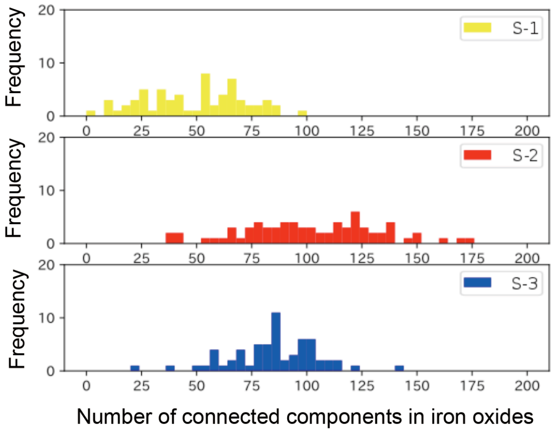

Fig. S7 Histograms of the number of connected components in iron oxide images.

We can understand the problem better by counting only small or large connected components. Figure S8 includes histograms showing the number of small and large connected components of iron oxide. In this analysis, we count the number of birth-death pairs whose birth value is larger than -7 (resp. less than -7) and whose death value is positive to count small (resp. large) connected components. These figures show that the number of small connected components of iron oxide increases from S-1 to S-2, whereas the number of large connected components does not change as the reduction process progresses.

However, a naive analysis using the number of connected components is not appropriate for calcium ferrites. Figure S9 shows histograms of the number of connected components, which gives no insight about the characteristic features of calcium ferrites during the reaction process.

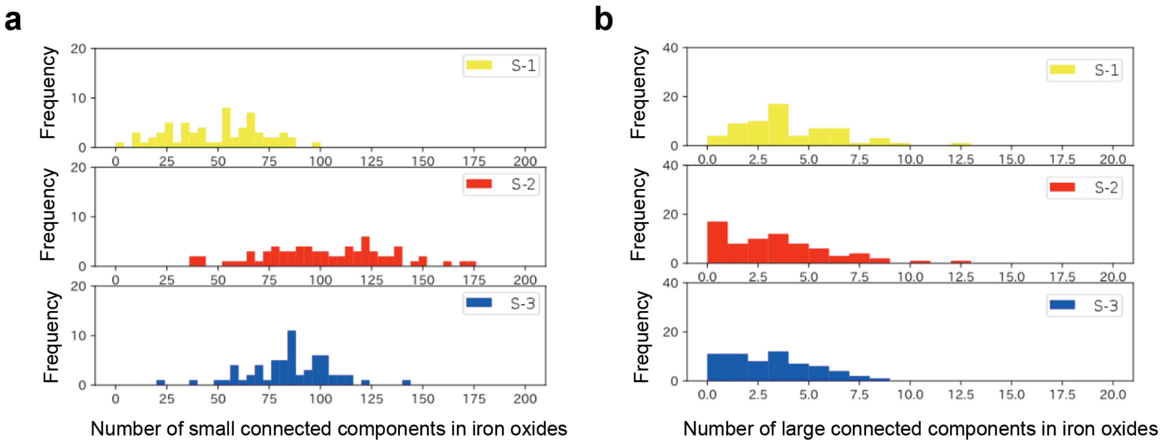

Fig. S8 Histograms of the number of connected components in iron oxide images. (**a**) small and (**b**) large ones.

These performance comparisons of our approach to simple image analysis showed that we can apply it as a simpler descriptor and those histograms provide us with some partial understandings of the reaction process, once we notice the fact that the number of small connected components of iron oxide increased from S-1 to S-2. However, we emphasize again that this finding was only clarified by the PCA on PDs and it is difficult to know it without any prior knowledge on the reaction mechanism. This comparison highlights an advantage of our method using machine learnings on persistent homology, which automatically captures significant features in the data-driven way.

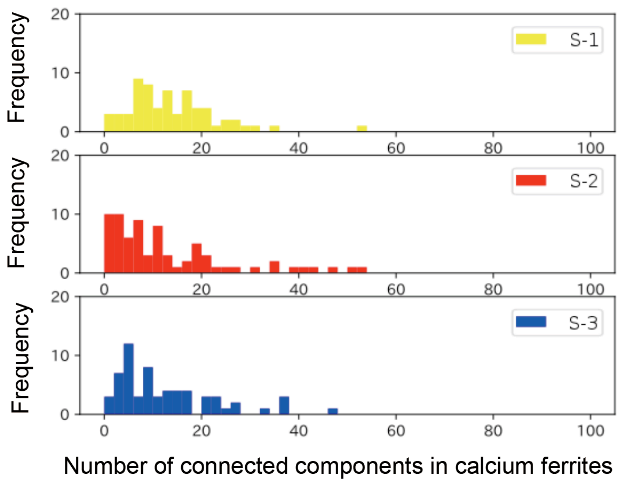

Fig. S9 Histograms of the number of connected components in calcium ferrites images.

We also compared the proficiency of our method to that of a standard machine learning method bag-of-keypoints approach with SIFT feature in the task of classifying S-1 and S-2 images. We selected 60 ×2 iron oxide images from S-1 and S-2. For each image, 400 keypoints (20 × 20) are prepared on a lattice, SIFT features are computed on each keypoint, and the feature dictionary is computed by *k*-means. Then, linear logistic regression with an l^1 penalty is applied to the constructed bag-of-keypoints histograms. We remark that, although nonlinear kernel is often used for simple classification tasks, the linear method with an l^1 penalty is more suitable for applying feature selection techniques to identify significant geometric features. Here, the parameters are adjusted by cross-validation. The accuracy rate computed by cross-validation was 75%, while that computed by our framework using linear logistic regression on persistence images was 80%.

Fig. S10 shows the result of feature selections using by the bag-of-keypoints approach with SIFT, where the blue (resp. red) circles correlate to S-1 (resp. S-2). The bag-of-keypoints analysis shows that the detected areas are somehow correlated to S-1 and S-2 but does not explicitly identify the geometric structures used to distinguish between S-1 and S-2. In contrast, our method provides a more explicit and intuitive understanding of the data. These results lead us to conclude that persistent homology analysis is more suitable than bag-of-keypoints analysis for processing images of materials.

a **b**

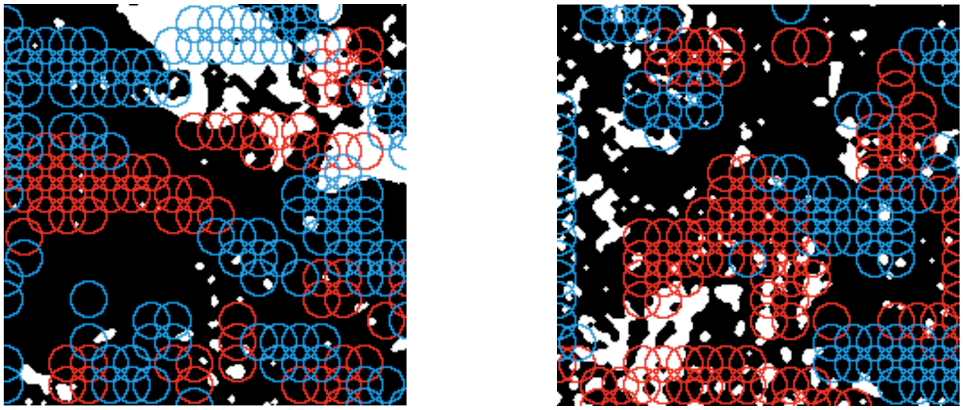

Fig. S10 Result of future selections by bag-of-keypoints with SIFT features. (**a**) and (**b**) correspond to S-1 and S-2, respectively. The blue (resp. red) circles correlate to S-1 (resp. S-2). Intuitively, the more the numbers of blue (resp. red) circles in the image is, the grater the likelihood the image is be S-1 (resp. S-2).

LASSO analysis. Linear regression is a statistical approach for estimating the relationship between a vector (i.e. an explanatory variable v_i) and a scalar value (i.e. a dependent variable s_i). The input of the linear regression is given by $\{(v_i, s_i) \in \mathbb{R}^n \times \mathbb{R}\}_{i=1}^{N}$, and is modeled as

$$s_i = a^T v_i + b + (\text{noise}), \tag{S5-4}$$

where the coefficient vector $a \in \mathbb{R}^n$ and the intercept $b \in \mathbb{R}$ are unknown parameters estimated from the input data. A standard linear regression method estimates a and b by the least-squares minimization. Furthermore, to avoid the overfitting problems of the analyses, we often combine this approach with the LASSO technique[13], in which the following cost function is minimized with respect to a and b:

$$\frac{1}{N}\sum_{i=1}^{N}\left\|a^T v_i + b - s_i\right\|^2 + \alpha\|a\|_1, \tag{S5-5}$$

where the term $\|a\|_1 = \sum_{j=1}^{n}\left|\mathrm{a}_j\right|$ is called the regularization term and $\alpha > 0$ is its controlling parameter.

One advantage of the LASSO regression is the sparseness of the learned vector a. In fact, by applying the LASSO to the vectors of the discretized PIs, we can identify a few grids (as a result of sparseness) in the histogram of the persistence diagram which have the largest impact on the dependent variable s_i because each element of the vector corresponds to a grid in the histogram.

In this study, we applied the LASSO method to detect the key birth–death pairs having the largest correlation with the areas (number of pixels) of the microcracks. To this end, the areas of the detected microcracks were used as dependent variables s_i in the LASSO regression. The parameter α was determined by cross-validation[12]. Then, four types of key birth–death pairs were identified, two for calcium ferrites (TS_{CF1} and TS_{CF2}) and two for iron oxides (TS_{IO1} and TS_{IO2}). These birth–death pairs are highly correlated with cracks and correspond to trigger sites.

Note S6. Datasets for PCA and linear regression

For this analysis, we prepared 80×3×4 X-CT image data from S-1, S-2, and S-3 for iron oxides, calcium ferrites, large pores, and cracks. The size of each image is 255×255 pixels (900 × 900 μm^2 in the real scale). Since an image with a large pore area has less information and is therefore harmful for statistical analysis, we remove those images from datasets. The threshold of the ratio of pore area is 0.6. Then, we use 64 × 4 images from S-1, 72 × 4 images from S-2, and 62 × 4 images from S-3. The resulting iron oxide and calcium ferrites images were used in the PCA. Only S-3 images were used in the LASSO analysis. Since the dataset is relatively small, to avoid overfitting, the parameter is adjusted by cross-validation for LASSO analysis.

References

1 Hamilton, J. D. G., Hoskins, B. F., Mumme, W. G., E., B. W. & A., M. M. The crystal structure and crystal chemistry of Ca2.3Mg0.8Al1.5FSi1.1Fe8.3O20 (SFCA): Solid solution limits and selected phase relationships of SFCA in the SiO2-Fe2O3-CaO(-Al2O3) system. *N. Jb. Miner. Abh.* **161** (1989).

2 Mumme, W. G., Clout, J. M. F. & Gable, R. W. The crystal structure of SFCA-I, $Ca_{3.18}Fe^{3+}_{14.66}Al_{1.34}Fe^{2+}_{0.82}O_{28}$, a homologue of the aenigmatite structure type, and new crystal structure refinements of ß-CFF, $Ca_{2.99}Fe^{3+}_{14.30}Fe^{2+}_{0.55}O_{25}$ and Mg-free SFCA, $Ca_{2.45}Fe^{3+}_{9.04}Al_{1.74}Fe^{2+}_{0.16}Si_{0.6}O_{20}$. *N. J. Miner. Abh.* **173**, 93-117, doi:0077-7775/98/0173-0093 (1998).

3 Kimura, M. *et al. In situ* observation of reduction kinetics and 2D mapping of chemical state for heterogeneous reduction in iron-ore sinters. *J. Phys. Conf. Ser.* **712**, 012077-012081, doi:10.1088/1742-6596/712/1/012077 (2016).

4 Igarashi, N. *et al.* New high-brilliance beamline BL-15A of the Photon Factory. *J. Phys. Conf. Ser.* **425**, 072016-072019 (2013).

5 Igarashi, N. *et al.* Newly designed double surface bimorph mirror for BL-15A of the photon factory. *AIP Conf. Procd.* **1741**, 040021 (2016).

6 Edelsbrunner, H., Morozov, D. & Pascucci, V. Persistence-sensitive simplification functions on 2-manifolds. *Proceedings of the Twenty-Second Annual Symposium on Computational Geometry*, 127-134, doi:10.1007/s00454-002-2885-2 (2006).

7 Zomorodian, A. & Carlsson, G. Computing persistent homology. *Discret. Comput. Geom.* **33**, 249-274, doi:10.1007/s00454-004-1146-y (2005).

8 Hiraoka, Y. *et al.* Hierarchical structures of amorphous solids characterized by persistent homology. *Proc. Natl. Acad. Sci.* **113**, 7035-7040, doi:10.1073/pnas.1520877113 (2016).

9 Saadatfar, M., Takeuchi, H., Robins, V., Francois, N. & Hiraoka, Y. Pore configuration landscape of granular crystallization. *Nat. Commun.* **8**, 15082, doi:10.1038/ncomms15082 (2017).

10 DIPHA (A Distributed Persistent Homology Algorithm). *Available at* https://github.com/DIPHA/dipha (2014).

11 Adams, H. *et al.* Persistence Images: A stable vector representation of persistent homology. *Journal of Machine Learning Research* **18**(11), 1-35 (2017).

12 Bishop, C. M. *Pattern recognition and Machine Learning* (Springer, 2006).

13 Tibshirani, R. Regression shrinkage and selection via the lasso. *J. Royal Stat. Soc. Ser. B (Methodol.)*, 267-288 (1996).
